# Understanding the Association Between Mental Health and Hair Loss

**DOI:** 10.7759/cureus.84777

**Published:** 2025-05-25

**Authors:** Mauri Malta, German Corso

**Affiliations:** 1 Dermatology, Saint James School of Medicine, Arnos Vale, VCT; 2 Child and Adolescent Psychiatry, Pediatric Psychiatry, Tropical Texas Behavioral Health, Harlingen, USA

**Keywords:** alopecia areata, androgenetic alopecia, body dysmorphic disorder, hair loss, mental health, psychodermatology, psychoneuroimmunology, stress-induced hair loss, telogen effluvium, trichotillomania

## Abstract

This literature review aims to analyze the association between mental health disorders and various types of hair loss, including telogen effluvium, androgenetic alopecia, alopecia areata, and compulsive disorders such as trichotillomania. A comprehensive review of recent studies was conducted to explore the bidirectional relationship between psychiatric conditions and hair loss, with emphasis on neurobiological mechanisms and psychosocial consequences. Findings show that psychiatric disorders can contribute to or exacerbate hair loss, while hair loss may lead to psychological symptoms such as anxiety, depression, and body dysmorphic disorder. Proposed mechanisms include immune dysfunction, neuroendocrine imbalance, microinflammation, brain-derived neurotrophic factor (BDNF) depletion, gut-brain-skin axis dysregulation, and medication-induced disruptions in hair cycling. Furthermore, individuals with somatic symptom disorder may report hair loss in the absence of clinical findings, complicating diagnosis and care. This review concludes that an interdisciplinary treatment model integrating dermatological and psychiatric support is essential for accurate diagnosis, effective treatment, and overall patient well-being.

## Introduction and background

In recent years, there has been a growing awareness of how mental health intersects with dermatological conditions, particularly those with visible and emotionally charged outcomes, such as hair loss [1]. What was once viewed predominantly as a cosmetic nuisance is now increasingly recognized as a legitimate clinical concern with psychological consequences. Conditions such as telogen effluvium, androgenetic alopecia, and alopecia areata often coexist with emotional distress, which may manifest as reduced self-confidence, social withdrawal, or broader psychological impairment [2].

Notably, this relationship appears to be bidirectional. On the one hand, psychological stress can serve as a trigger or aggravating factor for various types of hair loss. On the other hand, the experience of losing hair, especially in socially sensitive areas such as the scalp or eyebrows, can amplify psychiatric symptoms, such as anxiety and depression [3]. This dynamic is not limited to younger populations. Age-related conditions such as senescent alopecia also carry a psychosocial weight, particularly among older adults who may interpret hair thinning as a visual cue of aging or loss of vitality.

What is perhaps more concerning is that specific psychiatric disorders have been shown to directly influence how individuals perceive and respond to hair-related changes. Trichotillomania, for example, is not just a habit; it reflects an underlying pattern of anxiety and impulse control disruption. Similarly, disorders such as body dysmorphic disorder (BDD), major depressive disorder, and generalized anxiety disorder are frequently associated with perceived hair loss and negative self-image [4]. In fact, for many patients, psychological distress is the primary complaint, often overshadowing the actual dermatologic findings.

There are also compelling biological pathways that help bridge this mind-hair connection. Dysregulation of the hypothalamic-pituitary-adrenal (HPA) axis, which elevates systemic cortisol levels, can disrupt the normal hair cycle [5]. Additionally, recently, research into the gut-brain-skin axis has added a new layer of understanding, linking alterations in the microbiome with both mood disorders and scalp health.

The picture becomes even more complex when pharmacology is factored in. Medications such as selective serotonin reuptake inhibitors (SSRIs), lithium, and certain antiepileptics are well-documented contributors to medication-induced telogen effluvium. In parallel, some patients, especially those with somatic symptom disorder, may report hair loss in the absence of any physical explanation, highlighting the need for psychiatric insight in dermatologic practice.

This review brings together current findings on the psychological dimensions of hair loss, from age-related patterns to neurobiological underpinnings. More importantly, it emphasizes the value of integrated care, encouraging collaboration between dermatology and psychiatry to address both the physical presentation and the emotional burden of hair-related conditions [1].

## Review

Methods

This narrative review was conducted to explore the relationship between mental health problems and hair loss by systematically analyzing existing literature. A structured search was carried out across four major electronic databases: PubMed, PsycINFO, Scopus, and Web of Science, covering the period from 1992 to 2024. The search strategy utilized both controlled vocabulary (MeSH terms) and free-text keywords. Terms included the following: “hair loss”, “telogen effluvium”, “androgenetic alopecia”, “alopecia areata”, “trichotillomania”, “body dysmorphic disorder”, “psychoneuroimmunology”, and “mental health”. Boolean operators (“AND”, “OR”) were used to broaden or refine the search results as appropriate.

Following duplicate removal, a total of 83 articles were initially identified. Titles and abstracts were independently screened according to predefined criteria.

To determine eligibility for this review, studies had to meet several criteria. Only peer-reviewed articles, including original research, systematic reviews, and meta-analyses, were considered. These works needed to specifically explore the psychiatric factors contributing to hair loss, the mental health consequences associated with it, the biological pathways connecting the two, or propose models of integrated care. The analysis was limited to studies involving human subjects and published in English. We prioritized articles from international journals indexed in reputable databases such as PubMed, Scopus, and Web of Science to ensure high scientific quality and relevance. Preference was given to studies that included diverse demographic populations when available.

On the other hand, studies that centered exclusively on scarring forms of alopecia, such as lichen planopilaris, or those examining inherited hair loss syndromes without any psychological dimension, were excluded. Additionally, literature types that do not typically contribute substantial empirical evidence, such as opinion pieces, editorial commentary, brief conference summaries, or case studies, were left out. Articles were also disqualified if they lacked methodological strength or could not be fully accessed in their original format.

From the initial 83 articles, 25 were excluded after title and abstract screening due to irrelevance or failure to meet the inclusion criteria. The remaining 58 articles underwent full-text review. Following this assessment, 13 articles were excluded for the following reasons: four lacked a direct psychiatric-hair loss connection, four focused solely on genetic or scarring hair loss, three were editorial/commentary papers or case reports, and two failed to meet methodological quality standards or had inaccessible full text.

Ultimately, 45 articles were included in the final qualitative analysis. Selection was based not only on the scientific rigor of each study but also on its clinical relevance and alignment with the central aims of this review. Preference was given to literature demonstrating a bidirectional relationship between psychological conditions and hair loss, providing insight into how each can influence the other. Additional emphasis was placed on studies exploring physiological mechanisms - particularly those involving dysregulation of the HPA axis, immune imbalance, or neuroendocrine signaling. Studies that proposed integrative treatment models or encouraged collaboration between psychiatric and dermatological care were also prioritized for inclusion.

The structured search strategy and strict filtering process were designed to minimize selection bias and ensure that the evidence presented reflects the most reliable and current understanding of the psychodermatological interplay.

This study is a narrative literature review and did not involve the collection of new human or animal data. Therefore, approval from an institutional review board (IRB) and informed consent from participants were not required. All sources referenced were from publicly available, peer-reviewed publications, and proper credit has been given to the original authors to maintain academic integrity.

Results

Immune Dysregulation and Alopecia Areata

Alopecia areata offers a compelling lens to examine the effects of psychological stress on immune-mediated hair loss. In the setting of chronic emotional stress, the body’s neuroendocrine response, primarily through sustained activation of the hypothalamic-pituitary-adrenal (HPA) axis, leads to prolonged cortisol exposure. Over time, this hormonal state interferes with normal immune signaling and contributes to a breakdown in immune tolerance. One of the consequences is the loss of “immune privilege” in hair follicles, a specialized state that normally protects these structures from immune surveillance. Without this barrier, cytotoxic CD8⁺ T cells and natural killer (NK) cells can infiltrate the follicular environment, initiating localized autoimmune damage [6,7].

Beyond these cellular shifts, psychological stress appears to reshape cytokine dynamics in a way that favors inflammation. As Ahn et al. [8] described, chronic stress enhances both Th1 and Th17 immune responses, increasing the production of pro-inflammatory cytokines, such as interferon-gamma and interleukin-17. These molecules, in turn, contribute to the upregulation of MHC class I molecules and NKG2D ligands on follicular keratinocytes, making the follicles more visible and more vulnerable to immune attack. Simultaneously, stress-associated signaling may promote apoptotic pathways within follicular cells themselves, compounding the damage. These immunologic disruptions are not isolated phenomena; rather, they reflect a broader psychoneuroimmune interaction that links mental health stressors with immune dysfunction at the level of the skin.

Inflammatory and Oxidative Stress Pathways

**Figure 1:**
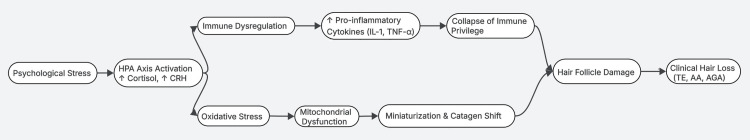
Psychobiological mechanisms linking psychological stress to hair loss. Chronic psychological stress activates the hypothalamic-pituitary-adrenal (HPA) axis, leading to elevated cortisol and corticotropin-releasing hormone (CRH) levels. This disrupts immune and oxidative balance, promoting pro-inflammatory cytokine release, immune privilege collapse, and mitochondrial dysfunction. These changes contribute to hair follicle miniaturization, early catagen transition, and eventual clinical hair loss, including telogen effluvium (TE), alopecia areata (AA), and androgenetic alopecia (AGA). Adapted and synthesized from Bertolini et al. [7]; Ahn et al. [8]; Arck et al. [12]; and Vallerand et al. [13] Image credit: Authors

Psychological stress not only affects immune balance, but it also initiates chronic low-grade inflammation and oxidative damage in the skin. Studies have shown that stress increases circulating levels of inflammatory mediators such as interleukin-1 (IL-1), tumor necrosis factor-alpha (TNF-α), and prostaglandins, all of which contribute to microinflammation around the hair follicle [9]. This environment disrupts the function of key follicular structures such as the dermal papilla and arrector pili muscle, pushing hair prematurely from the anagen (growth) phase into the telogen (shedding) phase. Prie al. [10] further demonstrated that oxidative stress exacerbated by chronic psychological load can impair mitochondrial function in follicular cells, accelerating their entry into the catagen phase and leading to miniaturization of hair shafts. Supporting this, Du et al. [11] have highlighted how oxidative stress modulates key pathways such as Wnt/β-catenin, mitogen-activated protein kinases (MAPK), and TGF-β, thereby altering the homeostasis and regeneration of hair follicles. These molecular events explain how conditions such as androgenetic alopecia and telogen effluvium (TE) may worsen under emotional strain. Figure 1 presents the psychobiological mechanisms linking psychological stress to hair loss.

Neurobiology of Trichotillomania (TTM) and Compulsive Behaviors

TTM is more than a behavioral compulsion; it is a psychiatric disorder rooted in neurobiological dysfunction, particularly in the circuits responsible for impulse regulation and habit formation. Neuroimaging research has consistently revealed abnormalities in the anterior cingulate cortex, dorsal striatum, and related frontostriatal pathways regions integral to monitoring behavior, suppressing urges, and modulating emotional responses [14,15]. Dysregulation in these areas may impair top-down control over motor actions, making individuals more susceptible to the involuntary, repetitive hair-pulling that characterizes TTM. Additionally, disruptions in neurotransmitter systems, especially involving serotonin and dopamine, likely contribute to the rewarding or tension-relieving sensations reported by patients following hair-pulling episodes, reinforcing the compulsive behavior through maladaptive reward processing. In some cases, these neurochemical and circuit-based disturbances overlap with those observed in obsessive-compulsive spectrum disorders, supporting the idea of shared mechanistic pathways [16].

TTM frequently coexists with other psychiatric conditions, particularly anxiety and BDD. In BDD, obsessive concern over hair appearance can manifest in compulsive grooming, concealment behaviors, or excessive mirror-checking patterns that not only aggravate physical symptoms but also amplify emotional distress [17,18]. These actions compound psychological impairment and further perpetuate hair damage.

Depression, Anxiety, and BDNF-Linked Hair Loss

Depression and anxiety frequently coexist with hair loss, especially in cases of TE and androgenetic alopecia. These associations are not purely circumstantial; they are supported by shared neurobiological pathways. Chronic stress and mood disorders are known to lower levels of brain-derived neurotrophic factor (BDNF), a protein involved in both emotional resilience and follicular health [19,20]. Reduced BDNF impairs the activity of dermal papilla cells, disrupts hair cycling, and contributes to follicular miniaturization. This connection suggests that strategies aimed at restoring BDNF through antidepressants, exercise, stress-reduction techniques, or certain nutritional supplements may benefit both psychological well-being and hair growth. Dai et al. [21] found that lifestyle interventions targeting BDNF levels were linked to improvements in both mood and follicular integrity.

Age-Related Hair Loss (Senescent Alopecia)

Hair thinning that occurs naturally with age, often referred to as senescent alopecia, is distinct from hormone-driven forms such as androgenetic alopecia. It typically begins around the sixth decade of life and reflects changes in the scalp’s biological environment. Studies have shown a gradual decline in stem cell renewal within hair follicles, reduced blood supply to the scalp, and lower levels of essential growth factors such as vascular endothelial growth factor (VEGF) and insulin-like growth factor-1 (IGF-1) [22,23]. These changes collectively shorten the hair growth phase and produce finer, less dense hair over time. While sometimes dismissed as a normal part of aging, this form of hair loss can still take a psychological toll. Older adults may feel less confident, avoid social situations, or struggle with body image, especially in societies that closely tie youth and appearance. In patients already coping with age-related transitions or emotional stressors, hair loss may further contribute to anxiety or depressive symptoms [24,25].

Gut-Brain-Skin Axis and Follicular Vulnerability

Emerging research points to a complex relationship between gut health, mental well-being, and skin function commonly referred to as the gut-brain-skin axis. Chronic psychological stress can alter the composition of gut microbiota, leading to increased intestinal permeability and systemic inflammation. These changes have downstream effects on skin immunity and scalp homeostasis, including the disruption of normal hair follicle cycling [26]. Gao et al. [27] demonstrated that microbial imbalance may interfere with immune regulation and hormonal signaling, increasing vulnerability to both psychological distress and hair disorders. Other studies suggest that restoring gut health through targeted nutrition, prebiotics, or probiotics may improve both mood and skin-related outcomes [28]. Although clinical evidence is still developing, this pathway offers promising insight into how internal stress responses can manifest externally, reinforcing the need for integrative approaches in psychodermatologic care.

Medication-Induced Hair Loss

**Figure 2:**
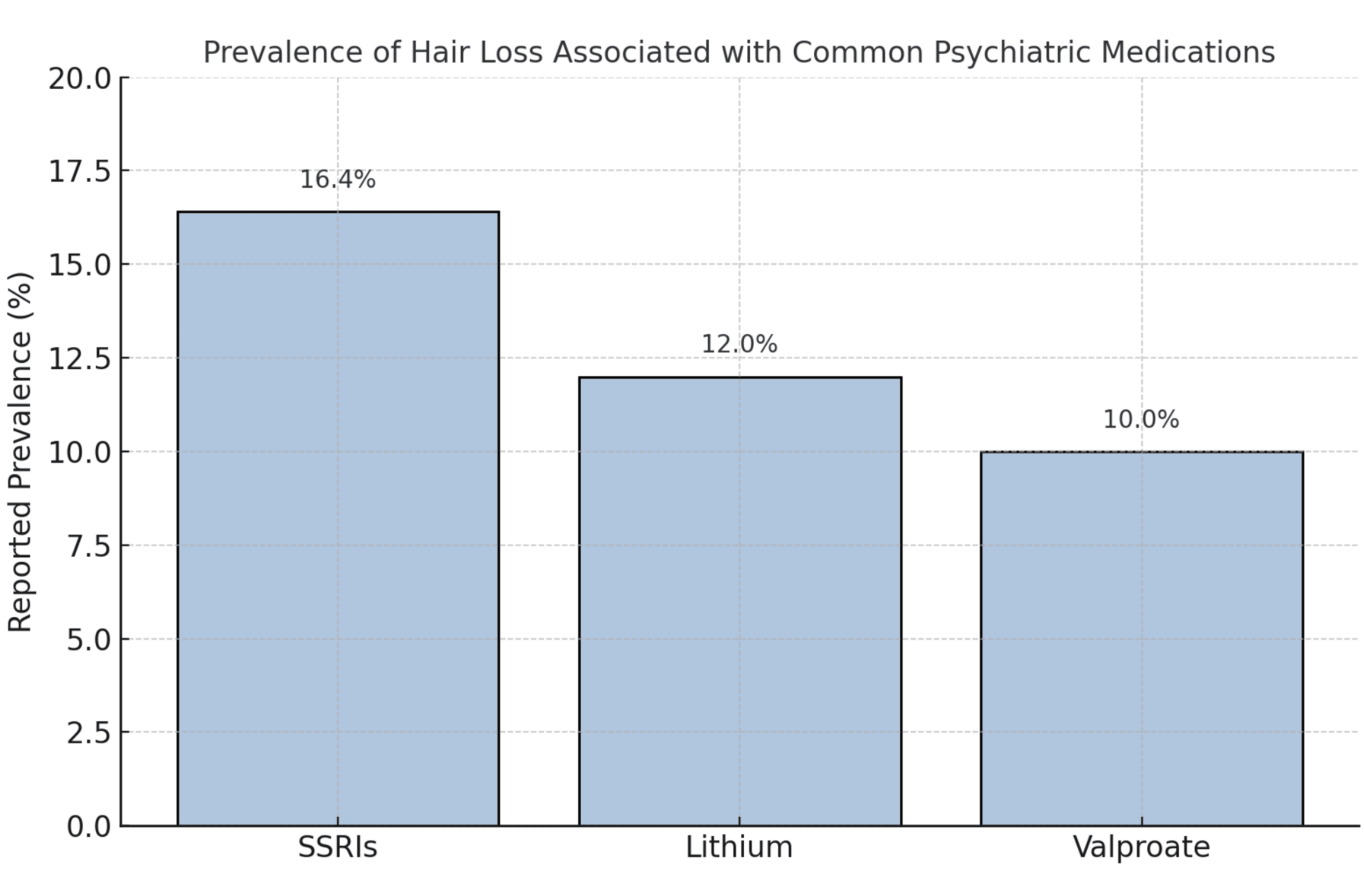
Prevalence of hair loss associated with common psychiatric medications. Observed rates of telogen effluvium and diffuse shedding vary across medications. SSRIs are associated with the highest reported prevalence (16.4%), followed by lithium (12%) and valproate (average 10%, ranging from 9% to 11%). These figures reflect the findings from observational and retrospective studies and underscore the need for clinicians to monitor dermatologic side effects in psychiatric care (Ezemma et al. [30]) Image credit: Authors

Certain psychiatric medications have been linked to increased hair shedding, most commonly in the form of TE. Among the most frequently reported are SSRIs, lithium, and valproate. These medications may interfere with the hair cycle by reducing the duration of the growth phase or triggering early follicular regression, potentially through mitochondrial stress or hormonal disruption [29]. In a large observational study, Ezemma et al. [30] found that 16.4% of individuals taking SSRIs long-term reported noticeable hair loss, compared to just 5.8% in a control group. Similarly, hair thinning has been observed in up to 12% of patients using lithium, and between 9% and 11% of those on valproate, especially at higher dosages [31]. Although often overlooked in clinical conversations, these side effects can be deeply distressing, particularly for patients already coping with mood disorders or body image concerns. Zhang et al. [32] noted that individual responses vary, with some patients more genetically predisposed to drug-induced shedding than others. Figure 2 presents the prevalence of hair loss associated with common psychiatric medications.

Somatic Symptom Disorder (SSD) and Subjective Hair Complaints

In some patients, complaints of hair loss occur despite normal clinical findings. This is often seen in individuals with SSD, where physical symptoms are driven by emotional distress rather than objective pathology. Patients may report increased shedding, fragility, or thinning, even though scalp examination reveals no signs of active alopecia. These subjective concerns are usually tied to heightened body preoccupation and are frequently accompanied by anxiety or depression [33]. Doerr et al. [34] found that individuals with SSD often exhibit elevated salivary cortisol, suggesting a state of sustained psychobiological stress. In many cases, these patients also engage in repetitive hair-checking, over-washing, or aggressive grooming, which can lead to mechanical damage over time.

COVID-19 and Hair Loss

The COVID-19 pandemic led to a noticeable rise in TE cases worldwide. While the virus itself was not the direct cause of hair loss in most patients, the pandemic triggered a combination of biological and psychological stressors that affected hair follicle cycling. On a physiological level, SARS-CoV-2 infection induces systemic inflammation and disrupts immune regulation, both of which are known to interfere with the normal hair growth process [35]. Elevated cytokines and acute stress hormones can push follicles prematurely into the shedding phase. At the same time, the emotional burden of the pandemic, including isolation, grief, financial uncertainty, and fear, created chronic activation of the HPA axis, raising cortisol levels and contributing to further follicular instability. Iancu et al. [36] emphasized that both the virus and the emotional trauma of the pandemic played roles in post-COVID hair loss, making TE in this context a marker of widespread psychophysiological disruption.

Psychosocial Burden and Quality of Life

Hair loss can significantly affect a person’s sense of identity, confidence, and social engagement, particularly when the condition is visible or resistant to treatment. Patients with conditions such as alopecia areata, androgenetic alopecia, or TE often report feelings of shame, isolation, and diminished self-worth, even when clinical severity is considered mild. These emotional reactions can be influenced by individual personality traits, including baseline anxiety, self-esteem, and coping style [37]. Supportive interventions such as guided self-image therapy, group counseling, or structured psychosocial programs have been shown to reduce emotional distress in patients dealing with chronic or unpredictable hair loss. These interventions have been shown to reduce emotional distress and may improve treatment outcomes for patients with visible hair loss [38].

Cultural Variability in Hair Loss Perception

The psychological response to hair loss is shaped not only by individual personality but also by cultural beliefs about appearance, aging, and gender. In many Western societies, a full head of hair is often associated with attractiveness, vitality, and social status. As a result, individuals experiencing hair thinning or baldness may internalize feelings of inadequacy or face stigma, especially women, who are more likely to experience social judgment tied to physical appearance [39]. However, this is not universal. In some cultures, hair loss is normalized or even respected, viewed as a natural part of aging or a symbol of wisdom. These variations influence how individuals interpret their condition and how likely they are to seek treatment or report emotional distress. Recognizing these cultural differences allows clinicians to provide more personalized, empathetic care and helps ensure that patient expectations and concerns are addressed in a way that feels relevant and respectful [38].

The evidence presented in this literature review highlights a multidimensional link between psychological stress, neuroendocrine function, immune dysregulation, and hair follicle health. These systems interact in complex ways, creating both direct and indirect pathways through which emotional distress can contribute to hair loss. Table 1 summarizes the major biological and psychological mechanisms discussed, offering a visual model of how chronic stress may drive follicular damage across various forms of alopecia.

**Table 1:** Summary of key physiological and psychological mechanisms linking mental health conditions with hair loss types. Each mechanism reflects a distinct interaction between biological pathways and emotional or behavioral factors described in the review.

Mechanism/Condition	Core Insight
Alopecia Areata (Autoimmune)	Stress collapses immune privilege via CD8+ T cells & HPA axis
Scalp Inflammation (Stress-Induced)	Cytokine surge (IL-1, TNF-α), oxidative stress damages the follicular support
Trichotillomania (TTM)	Impaired impulse control linked to brain regions managing habits and emotion
Depression & Anxiety	BDNF reduction impairs emotional resilience and follicle health
Senescent Alopecia	Age-related decline in stem cell activity, miniaturization, and oxidative damage
Medication-Induced Hair Loss	Psychotropics disrupt the hair cycle; some individuals genetically predisposed
Somatic Symptom Disorder (SSD	Hair concerns with no objective findings; linked to high cortisol
COVID-19-Related TE	Stress and inflammation from the virus and the pandemic trigger telogen effluvium
Gut-Brain-Skin Axis	Dysbiosis impairs immune and hormonal signaling, affecting hair follicles

Discussion

Understanding the intersection between psychiatric health and dermatological conditions, particularly hair loss, has become an increasingly important focus in clinical research. Often dismissed as a cosmetic concern, hair loss can have profound psychological consequences, triggering or worsening conditions such as anxiety, depression, and BDD. Psychiatric illness can, in turn, provoke or exacerbate hair loss through behavioral, neuroendocrine, and immunological mechanisms. This literature review reveals a complex, bidirectional relationship between mental health and hair disorders, reinforcing the importance of integrative, multidisciplinary care models. The following discussion synthesizes the physiological and psychological findings and explores their implications for clinical practice.

To begin, immune dysregulation plays a central role in alopecia areata, particularly when influenced by psychological stress. Chronic emotional strain interferes with immune privilege at the hair follicle, enabling CD8^+^ T cells and NK cells to target follicular structures [6,7]. Ahn et al. [8] further demonstrated that psychological stress enhances Th1 and Th17 responses, while neuroendocrine mediators such as substance P and CRH compromise immune tolerance [40]. These mechanisms collectively illustrate how psychological distress can provoke autoimmune hair loss. From a clinical standpoint, this reinforces the importance of considering both emotional health and immune function in the management of alopecia areata. Treatment plans that integrate psychological support alongside immunotherapies may improve outcomes by addressing both the physical and emotional components of disease activity. Interventions aimed at restoring neuroendocrine balance, such as cognitive-behavioral therapy, regular exercise, and sleep optimization, have demonstrated potential in reversing these effects [41,42]. As this field evolves, its integration into psychodermatologic practice may pave the way for more precise and holistic treatment strategies.

In addition to immune-mediated mechanisms, inflammatory and oxidative pathways contribute significantly to conditions such as androgenetic alopecia and TE. Chronic stress increases IL-1, TNF-α, and prostaglandin production, fostering a pro-inflammatory scalp environment that accelerates follicular transition into the telogen phase [9]. Prie et al. [10] also identified mitochondrial dysfunction as a compounding factor, reducing ATP output and heightening cellular vulnerability to stress-induced follicular miniaturization. These observations underscore the therapeutic value of combining anti-inflammatory strategies with interventions that reduce psychological stress. Clinically, the link between stress, inflammation, and mitochondrial dysfunction supports combining anti-inflammatory treatments with psychological support and lifestyle-based stress management to improve follicular resilience and reduce symptom recurrence.

Moving into compulsive and behavioral conditions, TTM and BDD exemplify how psychiatric symptoms can manifest directly through hair-focused behaviors. Neuroimaging studies link TTM to abnormalities in the anterior cingulate cortex and basal ganglia, affecting impulse control [13]. Simultaneously, patients with BDD may engage in repetitive grooming due to distorted self-perception and underlying anxiety [12]. From a clinical perspective, successful management of TTM and related disorders requires a multidisciplinary approach. Psychiatric assessment and behavioral therapies, particularly habit-reversal training and cognitive-behavioral therapy, are essential tools to help patients manage compulsive behaviors and reduce the risk of recurrence.

Further evidence supports a strong association between affective disorders and hair loss. Depression and anxiety frequently co-occur with TE and androgenetic alopecia, sharing dysregulated cortisol pathways and depleted BDNF as common denominators [19-21]. BDNF deficiency impairs dermal papilla cell function and hinders follicular regeneration. Therefore, interventions that enhance BDNF levels, whether pharmacologic, psychotherapeutic, or lifestyle-based, may offer dual benefits for both psychological and dermatologic outcomes [43]. Clinically, this underscores the value of holistic treatment models that integrate psychiatric care with dermatologic support, addressing the full scope of patient needs.

Age-related hair loss, or senescent alopecia, introduces additional biological and psychological considerations. Unlike androgenetic alopecia, senescent alopecia is characterized by diffuse thinning without a distinct pattern and occurs independently of androgenic activity, typically manifesting after the age of 50. Mechanistically, it is associated with decreased follicular stem cell activity, reduced vascular supply, and a decline in growth factors such as IGF-1 and VEGF [22,23]. From a psychosocial perspective, older adults may experience lowered self-esteem or social withdrawal in response to progressive hair thinning, especially in societies that equate youth with beauty and vitality [24,25]. For these reasons, senescent alopecia should be approached with the same clinical sensitivity as other hair disorders, with attention to both physical changes and emotional well-being.

The gut-brain-skin axis introduces another dimension to psychodermatology. Stress-induced dysbiosis impairs gut integrity, promoting systemic inflammation and altering hormonal signaling pathways, which can negatively affect both mood and scalp health [26,44]. Preliminary findings suggest that probiotics and dietary modification may offer dual benefits, supporting microbiome balance and reducing stress-related hair shedding [27]. Although clinical evidence is still developing, this pathway offers promising insight into how internal stress responses can manifest externally, reinforcing the need for integrative approaches in psychodermatologic care.

Psychiatric treatment itself may sometimes become a contributing factor to hair loss. SSRIs, lithium, and valproate are known to disrupt hair cycling, with reported TE rates between 9% and 16% [30,31]. These agents may prematurely terminate the anagen phase or impact follicular metabolism. Zhang et al. [32] emphasized that individual genetic variation can further influence susceptibility. These findings underline the importance of early identification and open communication. When patients are informed of potential side effects and supported in adjusting their treatment plan, they are more likely to remain adherent and emotionally stable throughout the course of psychiatric care.

SSD adds a unique layer of diagnostic and therapeutic challenge in cases of unexplained hair loss. These individuals often report significant distress and preoccupation with perceived hair changes despite objective dermatologic normalcy. Emerging evidence suggests that dysregulation of the HPA axis, particularly sustained elevations in cortisol, may contribute to heightened somatic focus and emotional amplification [33,34]. Functional neuroimaging studies have also implicated aberrant activity in regions governing emotional salience and interoceptive processing, such as the anterior insula and amygdala. Clinically, simple reassurance tends to fall short. Instead, effective management typically requires an integrated approach involving psychiatric consultation, cognitive-behavioral strategies, and psychoeducation to address the underlying affective drivers and reduce the risk of chronic disability.

This mind-body connection was further illuminated during the COVID-19 pandemic, which brought both biological and psychological stressors to the forefront. SARS-CoV-2 infection triggered immune dysregulation, while the psychological toll of grief, isolation, and fear activated the HPA axis, contributing to widespread cases of TE [35,36]. This reinforces the need to approach post-COVID hair loss with both medical evaluation and mental health support. Addressing the emotional impact alongside physical symptoms can help patients better understand the temporary nature of the condition and improve adherence to treatment and follow-up.

Psychological stress is increasingly recognized as a key factor in the pathogenesis of various hair disorders. Research shows that stress-induced oxidative damage and inflammatory cytokine activity can disrupt hair follicle cycling and mitochondrial function, leading to early follicle regression and shedding [11]. While much of this evidence comes from Western cohorts, emerging data from developing countries also highlight similar trends under different societal stressors. For instance, a multicenter study conducted in Saudi Arabia found that patients with alopecia areata experienced significant psychological impacts, including anxiety and depression, underscoring the need for comprehensive care approaches that address both dermatological and psychological aspects of the condition [45]. Similarly, research from Nepal reported high prevalence rates of depression and anxiety among alopecia areata patients, emphasizing the psychological burden of the disease in diverse cultural contexts [45].

Cultural frameworks play a critical role in shaping how individuals interpret and emotionally respond to hair loss. In many Western contexts, hair thinning or baldness is often perceived through a lens of aesthetic loss, aging, or diminished social value, whereas in other cultures, it may be viewed as a neutral feature or even symbolically linked to wisdom, spiritual purity, or social status. These culturally embedded narratives influence not only the degree of psychological distress but also the likelihood of seeking treatment. Clinicians who remain attuned to such cultural dimensions are better equipped to deliver care that is both empathetic and contextually appropriate, fostering more meaningful communication and alignment with patient expectations [38,39].

Strengths and limitations

This review brings together a broad and interdisciplinary range of perspectives to better understand the connection between psychiatric disorders and hair loss. One of its key strengths is the way it integrates research across dermatology, psychiatry, immunology, and neurobiology fields that are often considered separately in clinical practice. By bridging these disciplines, the review sheds light on both the emotional and biological drivers behind hair loss, including immune system shifts, hormonal changes, neurotrophic factors, and gut-brain interactions. Another strength lies in the study’s focus on recent literature, capturing up-to-date findings that reflect emerging patterns in patient care and scientific understanding. The inclusion of both behavioral and molecular mechanisms gives the discussion depth and allows for a more holistic view of treatment approaches. For clinicians, this provides practical insights into how psychological factors can influence dermatological conditions while offering researchers a structured foundation for future study.

As this review adequately details, it comes with limitations. The search was restricted to studies published between 1992 and 2025, which helped focus the analysis on contemporary evidence but may have excluded older studies that could offer historical context or long-term trends. The review also excluded genetic and scarring forms of alopecia, narrowing the focus to stress-responsive and psychiatric-associated hair loss. While this decision was intentional to maintain thematic consistency, it may have left out important findings related to genetic predisposition and chronic inflammatory scalp conditions. Language was another limiting factor; only English-language articles were reviewed, meaning that research published in other languages was not included, even if potentially relevant. Although the databases used were comprehensive, some closed-access or non-indexed studies might not have been captured. Additionally, the review acknowledges that individual variability plays a large role in clinical outcomes. Personality traits, lifestyle, and social context can all influence the way patients experience and cope with hair loss, and future studies should aim to explore these factors with more personalized approaches and diverse populations.

## Conclusions

This literature review underscores that hair loss is not a superficial or solely cosmetic concern but a complex psychodermatological condition shaped by stress-related immune dysregulation, neuroendocrine shifts, and psychological comorbidities. These interconnected factors call for collaborative care involving dermatologists, psychiatrists, and primary providers who can address both the biological drivers and emotional consequences of hair disorders.

Emerging insights into the gut-brain-skin axis, BDNF pathways, and somatic symptom presentations offer new directions for personalized interventions. At the same time, underrecognized contributors such as psychiatric medications or cultural interpretations of hair loss demand more attention in both research and clinical practice. Going forward, treatment models must move beyond symptom management to embrace holistic strategies rooted in education, integrated diagnostics, and mental health screening. By aligning dermatologic care with psychological insight, providers can improve outcomes and enhance quality of life for individuals navigating the dual burden of hair loss and emotional distress.

## References

[1] Moattari CR, Jafferany M (2022). Psychological aspects of hair disorders: consideration for dermatologists, cosmetologists, aesthetic, and plastic surgeons. Skin Appendage Disord.

[2] Macbeth AE, Holmes S, Harries M (2022). The associated burden of mental health conditions in alopecia areata: a population-based study in UK primary care. Br J Dermatol.

[3] Jafferany M, Patel A (2020). Trichopsychodermatology: the psychiatric and psychosocial aspects of hair disorders. Dermatol Ther.

[4] Dhami L (2021). Psychology of hair loss patients and importance of counseling. Indian J Plast Surg.

[5] Grymowicz M, Rudnicka E, Podfigurna A, Napierala P, Smolarczyk R, Smolarczyk K, Meczekalski B (2020). Hormonal effects on hair follicles. Int J Mol Sci.

[6] Tzur Bitan D, Berzin D, Kridin K, Cohen A (2022). The association between alopecia areata and anxiety, depression, schizophrenia, and bipolar disorder: a population-based study. Arch Dermatol Res.

[7] Bertolini M, McElwee K, Gilhar A, Bulfone-Paus S, Paus R (2020). Hair follicle immune privilege and its collapse in alopecia areata. Exp Dermatol.

[8] Ahn D, Kim H, Lee B, Hahm DH (2023). Psychological stress-induced pathogenesis of alopecia areata: autoimmune and apoptotic pathways. Int J Mol Sci.

[9] Sadick NS, Callender VD, Kircik LH, Kogan S (2017). New insight into the pathophysiology of hair loss trigger a paradigm shift in the treatment approach. J Drugs Dermatol.

[10] Prie BE, Voiculescu VM, Ionescu-Bozdog OB (2015). Oxidative stress and alopecia areata. J Med Life.

[11] Du F, Li J, Zhang S, Zeng X, Nie J, Li Z (2024). Oxidative stress in hair follicle development and hair growth: signalling pathways, intervening mechanisms and potential of natural antioxidants. J Cell Mol Med.

[12] Arck PC, Handjiski B, Hagen E, Joachim R, Klapp BF, Paus R (2001). Indications for a 'brain-hair follicle axis (BHA)': inhibition of keratinocyte proliferation and up-regulation of keratinocyte apoptosis in telogen hair follicles by stress and substance P. FASEB J.

[13] Vallerand IA, Lewinson RT, Parsons LM (2019). Assessment of a bidirectional association between major depressive disorder and alopecia areata. JAMA Dermatol.

[14] Torales J, Ruiz Díaz N, Ventriglio A (2021). Hair-pulling disorder (trichotillomania): etiopathogenesis, diagnosis and treatment in a nutshell. Dermatol Ther.

[15] Uhlmann A, Dias A, Taljaard L, Stein DJ, Brooks SJ, Lochner C (2020). White matter volume alterations in hair-pulling disorder (trichotillomania). Brain Imaging Behav.

[16] Grant JE, Chamberlain SR (2016). Trichotillomania. Am J Psychiatry.

[17] Phillips KA, Didie ER, Menard W, Pagano ME, Fay C, Weisberg RB (2006). Clinical features of body dysmorphic disorder in adolescents and adults. Psychiatry Res.

[18] Lochner C, Grant JE, Odlaug BL, Woods DW, Keuthen NJ, Stein DJ (2012). DSM-5 field survey: hair-pulling disorder (trichotillomania). Depress Anxiety.

[19] Mohamed NE, Soltan MR, Galal SA, El Sayed HS, Hassan HM, Khatery BH (2023). Female pattern hair loss and negative psychological impact: possible role of brain-derived neurotrophic factor (BDNF). Dermatol Pract Concept.

[20] Cheng Y, Lv LJ, Cui Y, Han XM, Zhang Y, Hu CX (2024). Psychological stress impact neurotrophic factor levels in patients with androgenetic alopecia and correlated with disease progression. World J Psychiatry.

[21] Dai YX, Tai YH, Chen CC, Chang YT, Chen TJ, Chen MH (2020). Bidirectional association between alopecia areata and major depressive disorder among probands and unaffected siblings: a nationwide population-based study. J Am Acad Dermatol.

[22] Deng Y, Wang M, He Y, Liu F, Chen L, Xiong X (2023). Cellular senescence: ageing and androgenetic alopecia. Dermatology.

[23] Inui S, Itami S (2011). Molecular basis of androgenetic alopecia: from androgen to paracrine mediators through dermal papilla. J Dermatol Sci.

[24] Cash TF (1992). The psychological effects of androgenetic alopecia in men. J Am Acad Dermatol.

[25] Williamson D, Gonzalez M, Finlay AY (2001). The effect of hair loss on quality of life. J Eur Acad Dermatol Venereol.

[26] Bowe WP, Logan AC (2011). Acne vulgaris, probiotics and the gut-brain-skin axis - back to the future?. Gut Pathog.

[27] Gao T, Wang X, Li Y, Ren F (2023). The role of probiotics in skin health and related gut-skin axis: a review. Nutrients.

[28] Yu P, Teng X, Liu T, Li Y, Ni J, Xue S, Wang J (2022). Effect of an oral probiotic formula on scalp and facial skin condition, glucose, and lipid metabolism. Funct Foods Health Dis.

[29] Kıvrak Y, Yağcı İ, Üstündağ MF, Özcan H (2015). Diffuse hair loss induced by sertraline use. Case Rep Psychiatry.

[30] Ezemma O, Devjani S, Jothishankar B, Kelley KJ, Senna M (2024). Drug-induced alopecia areata: a systematic review. J Am Acad Dermatol.

[31] McKinney PA, Finkenbine RD, DeVane CL (1996). Alopecia and mood stabilizer therapy. Ann Clin Psychiatry.

[32] Zhang D, LaSenna C, Shields BE (2023). Culprits of medication-induced telogen effluvium, part 1. Cutis.

[33] Hüfner K, Tymoszuk P, Sahanic S (2023). Persistent somatic symptoms are key to individual illness perception at one year after COVID-19 in a cross-sectional analysis of a prospective cohort study. J Psychosom Res.

[34] Doerr JM, Nater UM, Feneberg AC, Mewes R (2021). Differential associations between fatigue and psychobiological stress measures in women with depression and women with somatic symptom disorder. Psychoneuroendocrinology.

[35] Rivetti N, Barruscotti S (2020). Management of telogen effluvium during the COVID-19 emergency: psychological implications. Dermatol Ther.

[36] Iancu GM, Molnar E, Ungureanu L, Șenilă SC, Hașegan A, Rotaru M (2023). SARS-CoV-2 infection—a trigger factor for telogen effluvium: review of the literature with a case-based guidance for clinical evaluation. Life (Basel).

[37] Russo PM, Fino E, Mancini C, Mazzetti M, Starace M, Piraccini BM (2019). HrQoL in hair loss-affected patients with alopecia areata, androgenetic alopecia and telogen effluvium: the role of personality traits and psychosocial anxiety. J Eur Acad Dermatol Venereol.

[38] Hunt N, McHale S (2005). The psychological impact of alopecia. BMJ.

[39] Hwang HW, Ryou S, Jeong JH (2024). The quality of life and psychosocial impact on female pattern hair loss. Ann Dermatol.

[40] Dhabhar FS (2014). Effects of stress on immune function: the good, the bad, and the beautiful. Immunol Res.

[41] O'Sullivan JD, Peters EM, Amer Y (2022). The impact of perceived stress on the hair follicle: towards solving a psychoneuroendocrine and neuroimmunological puzzle. Front Neuroendocrinol.

[42] Duman RS, Monteggia LM (2006). A neurotrophic model for stress-related mood disorders. Biol Psychiatry.

[43] Singh J, Vanlallawmzuali Vanlallawmzuali, Singh A (2024). Microbiota-brain axis: exploring the role of gut microbiota in psychiatric disorders - a comprehensive review. Asian J Psychiatr.

[44] Almulhim NA, Alojail HY, Aljughayman MA (2024). Awareness, beliefs, and psychological impact of patients with alopecia areata in Saudi Arabia: a multi-center study. Patient Prefer Adherence.

[45] Marahatta S, Agrawal S, Adhikari BR (2020). Psychological impact of alopecia areata. Dermatol Res Pract.

